# Blood pressure variability plays a critical role in determining the prognosis of acute ischemic stroke combined with hypertension

**DOI:** 10.3389/fneur.2026.1834657

**Published:** 2026-05-05

**Authors:** Keqiong Yan, Lan Ye, Mei Wu, Jun Yang

**Affiliations:** 1 Department of Neurology, The First Affiliated Hospital of Chongqing Medical University, Chongqing, China; 2 Department of Pediatrics, Huashan Hospital, Fudan University, Shanghai, China

**Keywords:** acute ischemic stroke, blood pressure variability, hypertension, prognosis, risk factor

## Abstract

**Background:**

Individuals with hypertension exhibit high blood pressure variability (BPV), which is associated with an increased risk of acute ischemic stroke (AIS) and poor post-stroke outcomes. Therefore, managing BPV may help reduce the incidence of AIS and improve patient prognosis.

**Aim:**

This prospective cohort study investigated the effects of BPV regulation on the prognosis of patients with AIS and hypertension, using modified Rankin Scale (mRS) and National Institutes of Health Stroke Scale (NIHSS) scores as primary outcomes to inform individualized treatment strategies.

**Methods:**

Patients with AIS from December 2021 to June 2024 were included in the study and divided into two groups based on systolic BPV (SBPV) measured more than 24 h after symptom onset: the control group (SBPV 10–20%) and the observation group (SBPV outside 10–20%). The observation group received antihypertensive regimen adjustments based on 24-h ambulatory blood pressure monitoring (ABPM) during hospitalization, and the group was subsequently stratified into Groups 1 and 2 according to the improvement in SBPV assessed at the two-week outpatient follow-up after the intervention. Additionally, modified Rankin Scale (mRS) and National Institutes of Health Stroke Scale (NIHSS) scores were collected during the acute phase and again after 90 days.

**Results:**

A total of 75 patients were included. The observation group had higher acute-phase diastolic BPV (*p* < 0.05) and worse 90-day mRS and NIHSS scores (p < 0.05) than the control group. Despite similar baseline scores within the observation group, patients with improved SBPV (Group 1) showed better 90-day outcomes than those without improvement (Group 2) (*p* < 0.05).

**Conclusion:**

Despite the small sample, our findings suggest that BPV may serve as a prognostic marker for functional outcomes in AIS. Tailored antihypertensive therapy aimed at reducing BPV showed a preliminary association with improved patient prognosis.

## Introduction

Acute ischemic stroke (AIS), characterized by high incidence, recurrence, disability, and mortality rates, is the most prevalent cerebrovascular disease (1). In China, the incidence of stroke has been increasing annually, with new cases accounting for one-quarter of the global total. Among individuals aged 40 years and older, ischemic stroke constitutes 86.8% of all stroke cases in China (2). Predictions indicate that the annual number of new AIS cases could exceed two million, with a recurrence rate of up to 17.7%, placing a considerable burden on families and society (3, 4). Therefore, effective prevention strategies for stroke are urgently needed.

Hypertension is one of the most prevalent risk factors for AIS, affecting approximately 70% of patients (5, 6). With the increasing clinical use of 24-h ambulatory blood pressure monitoring (ABPM), greater attention has been directed toward the role of blood pressure variability (BPV) in AIS. BPV refers to fluctuations in blood pressure over time and is commonly quantified using standard deviation or coefficient of variation (7). Based on the nocturnal BP fall, individuals are classified into four categories: dippers (nocturnal fall 10–20%), non-dippers (0–10%), anti-dippers (negative day–night difference), and over-dippers (>20%) (8). Research has shown that the probability of cerebrovascular events was greater among non-dippers than dippers (9). Clinically, dippers (BPV within the 10–20% range) is considered normal. Numerous studies have shown that BPV, independent of mean BP, is a significant predictor of poor clinical outcomes in patients with AIS and an independent risk factor for cardiovascular and cerebrovascular diseases (10, 11). Higher BPV is associated with worse prognosis and increased risk of AIS (12).

National and international guidelines for cerebrovascular diseases explicitly recommend BP management for the prevention and treatment of AIS (13). However, prospective studies examining how modulation of BPV affects the incidence and prognosis of AIS remain limited. Therefore, this study aimed to modify antihypertensive regimens and explore the relationship between BPV and AIS outcomes. The findings may provide foundational evidence for individualized BP management to reduce the incidence and recurrence of AIS and improve patient prognosis.

## Materials and methods

### Study design

This single-center prospective cohort study enrolled patients with AIS and primary hypertension (including those with normalized BP after treatment) admitted to the Department of Neurology of the First Affiliated Hospital of Chongqing Medical University from December 2021 to June 2024. The inclusion criteria were: (1) individuals with AIS and primary hypertension; (2) completion of 24-h ABPM during hospitalization; and (3) provision of informed consent. The exclusion criteria were: (1) malignant hypertension uncontrolled by medicine; (2) confirmed secondary hypertension; (3) intracerebral hemorrhage; and (4) severe other systemic diseases, such as acute myocardial infarction, malignant tumors, and severe hematological disorders.

The Ethics Committee of the First Affiliated Hospital of Chongqing Medical University [Scientific Research Ethics (2023–261)] approved this study.

### 24-h dynamic BP monitoring

Patients were divided into two groups based on SBPV measured more than 24 h after symptom onset: the control group (SBPV 10–20%) and the observation group (SBPV outside 10–20%). Patients in the observation group received tailored adjustments to their antihypertensive therapy, primarily involving the addition or switch to calcium channel blockers such as amlodipine, based on ABPM results.

Follow-up ABPM was conducted in the outpatient department 2 weeks later. Based on the results, the observation group was further subdivided into Group 1 (SBPV improved, approaching the 10–20% range) and Group 2 (SBPV did not improve or deviated further). Neurological severity and functional outcomes were assessed using the NIHSS and mRS scores at admission and during an in-person follow-up at 90 days.

### Statistical analyses

SAS software (version 9.4; SAS Institute, Cary, NC, United States) was used for all statistical analyses. Normally distributed quantitative data are expressed as means ± standard deviations, and comparisons were made using independent sample t-tests. Data with skewed distributions were compared using the Wilcoxon rank-sum test. Median and interquartile spacing were used to characterize the measurement data. Examples and rates were used to describe the classification data, and chi-square tests were used for group comparisons. *p*-values of <0.05 were considered statistically significant.

## Results

### Control group versus observation group

**Figure 1:**
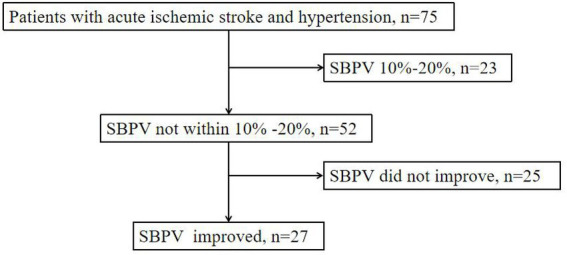
Flow chart of patient grouping. SBPV indicates systolic blood pressure variability.

After excluding patients with NIHSS >15 or missing ABPM data, a total of 75 patients (16 females and 59 males) were included (Figure 1); the average age was 64.45 ± 9.5 years. The control group consisted of 23 patients, and the observation group consisted of 52 patients. The average SBPV was 12.22% in the control group and 2.05% in the observation group. Table 1 presents the patient data.

**Table 1:** Clinical characteristics of the 75 patients recruited in this study.

Variable	Total	Control group (SBPV 10–20%, *n* = 23)	Observation group (SBPV outside 10–20%, *n* = 52)	*t*/*χ*2	*P*
Age	64.45 ± 9.5	66.3 ± 8.17	63.63 ± 10	1.124	0.265
Gender, Male, *n*(%)	59(78.67)	18(78.26)	41(78.85)	<0.001	0.999
Smoking, *n*(%)	40(53.33)	11(47.83)	29(55.77)	0.404	0.525
Drinking, *n*(%)	33(44.00)	9(39.13)	25(46.15)	0.319	0.572
Diabetes, *n*(%)	27(36.00)	10(43.48)	17(32.69)	0.805	0.370
BMI (kg/m^2^)	24.51 ± 2.53	24.49 ± 2.77	24.53 ± 2.44	−0.066	0.947
TC (mmol/L)	4.35 ± 1.02	4.05 ± 1.22	4.48 ± 0.9	−1.703	0.093
TG (mmol/L)	1.69 ± 0.89	1.78 ± 1.1	1.65 ± 0.79	0.589	0.558
LDL (mmol/L)	2.76 ± 0.96	2.49 ± 1.15	2.87 ± 0.85	−1.609	0.112
HDL (mmol/L)	1.07 ± 0.26	1.03 ± 0.23	1.09 ± 0.27	−0.868	0.388
DBPV in acute phase (%)	8.17 ± 7.7	14.11 ± 4.92	5.54 ± 7.25	5.966	**<0.001**
Average SBP (mmHg)	138.37 ± 13.34	136.65 ± 14.97	139.13 ± 12.64	−0.741	0.461
Average DBP (mmHg)	81.31 ± 11.02	78.96 ± 12.05	82.35 ± 10.49	−1.233	0.222

BMI indicates body mass index; TC, the total cholesterol, TG, triglyceride; LDL, low-density lipoprotein; HDL, high-density lipoprotein; DBPV, diastolic blood pressure variability; SBP, systolic blood pressure; DBP, diastolic blood pressure. Bold values indicate statistical significance (*p* < 0.05).

Diastolic BPV in the acute phase was significantly higher in the observation group than in the control group (*p* < 0.05). No significant differences were observed between groups in age, sex, diabetes, smoking history, alcohol consumption, body mass index, lipid profiles, or mean systolic and diastolic BP.

Both NIHSS and mRS scores were significantly worse in the observation group than in the control group during the acute phase and at 90 days (*p* < 0.05; Table 2).

**Table 2:** Comparison of severity and prognosis between the control group and the observation group.

Variable	Total	Control group (SBPV 10–20%, *n* = 23)	Observation group (SBPV outside 10–20%, *n* = 52)	*Z*	*P*
NIHSS in acute phase	2(1,3)	2(1,2)	2(1,3)	−2.411	**0.016**
mRS in acute phase	2(1,3)	2(1,3)	3(2,3)	−2.874	**0.004**
NIHSS after 90 days	1(0,1)	0(0,1)	1(0,2)	−2.755	**0.006**
mRS after 90 days	1(0,2)	0(0,1)	1(1,2)	−3.457	**0.001**

NIHSS indicates National Institutes of Health Stroke Scale; mRS, the modified Rankin Scale. Bold values indicate statistical significance (*p* < 0.05).

### Observation group 1 versus group 2

Within the observation group, 27 patients showed improved SBPV after intervention (Group 1), while 25 did not (Group 2). Baseline characteristics were similar between the two subgroups, except for diastolic BPV after intervention, which was significantly higher in Group 1 (*p* < 0.05; Table 3).

**Table 3:** Clinical characteristics of the 52 patients in observation group.

Variable	Total	Observation group 1 (SBPV improved, *n* = 27)	Observation group 2 (SBPV not improved, *n* = 25)	*t*/*χ*^2^	*P*
Age	63.63 ± 10	63.04 ± 9.84	64.28 ± 10.35	−0.444	0.659
Gender, Male, *n*(%)	41(78.85)	20(74.07)	21(84.00)	0.767	0.381
Smoking, *n*(%)	29(55.77)	13(48.15)	16(64.00)	1.322	0.250
Drinking, *n*(%)	24(46.15)	11(40.74)	13(52.00)	0.662	0.416
Diabetes, *n*(%)	17(32.69)	8(29.63)	9(36.00)	0.239	0.625
BMI (kg/m^2^)	24.53 ± 2.44	24.58 ± 2.15	24.47 ± 2.76	0.168	0.867
TC (mmol/L)	4.48 ± 0.9	4.37 ± 0.9	4.6 ± 0.89	−0.893	0.376
TG (mmol/L)	1.65 ± 0.79	1.57 ± 0.76	1.74 ± 0.83	−0.749	0.457
LDL (mmol/L)	2.87 ± 0.85	2.79 ± 0.84	2.96 ± 0.86	−0.705	0.484
HDL (mmol/L)	1.09 ± 0.27	1.05 ± 0.22	1.13 ± 0.32	−1.032	0.308
DBPV in acute phase (%)	5.54 ± 7.25	3.75 ± 7.47	7.48 ± 6.61	−1.897	0.064
Average SBP in acute phase (mmHg)	139.13 ± 12.64	136.85 ± 11.96	141.6 ± 13.12	−1.365	0.178
Average DBP in acute phase (mmHg)	82.35 ± 10.49	82.56 ± 8.45	82.12 ± 12.5	0.148	0.883
DBPV after intervention (%)	9.94 ± 7.38	12.76 ± 6.81	6.9 ± 6.84	3.097	**0.003**
Average SBP after intervention (mmHg)	129.31 ± 12.38	128.33 ± 10.15	130.36 ± 14.56	−0.586	0.561
Average DBP after intervention (mmHg)	78.06 ± 7.94	77.89 ± 6.36	78.24 ± 9.49	−0.155	0.877

BMI indicates body mass index; TC, the total cholesterol, TG, triglyceride; LDL, low-density lipoprotein; HDL, high-density lipoprotein; DBPV, diastolic blood pressure variability; SBP, systolic blood pressure; DBP, diastolic blood pressure. Bold values indicate statistical significance (*p* < 0.05).

No significant differences were found in acute-phase NIHSS and mRS scores between Groups 1 and 2 (*p* > 0.05). However, at 90 days, both NIHSS and mRS scores were significantly better in Group 1 than in Group 2 (*p* < 0.05; Table 4).

**Table 4:** Comparison of severity and prognosis between the observation group 1 and the observation group 2.

Variable	Total	observation group 1 (SBPV improved, *n* = 27)	observation group 2 (SBPV not improved, *n* = 25)	*Z*	*P*
NIHSS in acute phase	2(1,3)	2(1,4)	3(1,3)	−0.094	0.925
mRS in acute phase	3(2,3)	3(2,4)	3(2,3)	−0.810	0.418
NIHSS after 90 days	1(0,2)	1(0,1)	1(1,2)	−2.624	**0.009**
mRS after 90 days	1(1,2)	1(1,2)	2(1,3)	−2.313	**0.021**

NIHSS indicates National Institutes of Health Stroke Scale; mRS, the modified Rankin Scale. Bold values indicate statistical significance (*p* < 0.05).

## Discussion

The BPV is a clinically important parameter closely associated with target organ damage, the progression and prognosis of cardiovascular and cerebrovascular diseases, and BP control (14). Patients with hypertension often exhibit elevated BPV in addition to elevated mean BP (8).

Our findings are consistent with previous studies showing that BPV is an independent risk factor for stroke and influences the occurrence, progression, and severity of AIS (15, 16). Despite heterogeneity across studies—such as differences in population, sample size, methodology, and BPV measurement duration—accumulating evidence supports BPV as a major risk factor for AIS. In patients with AIS, elevated BPV is strongly correlated with mortality, stroke recurrence, and long-term functional recovery (17). However, variations in baseline BP, antihypertensive medications, follow-up duration, and confounding factors have led to inconsistent results. Further research is needed to identify the most prognostically relevant BPV indicators.

The mechanisms by which BPV influences AIS remain unclear. One hypothesis is that cerebral autoregulation maintains relatively constant perfusion within a certain BP range, but when BPV exceeds this capacity, stroke risk increases (18). Hemodynamic instability and high BPV may damage vessel walls, leading to endothelial dysfunction, intima-media thickening, atherosclerosis, and plaque formation (19). Activation of the renin-angiotensin system may also contribute by increasing angiotensin II and catecholamine levels, promoting smooth muscle hypertrophy and vascular stenosis (20). Elevated BPV may also trigger inflammatory responses, increasing cytokines such as interleukin-1β and tumor necrosis factor-*α*, which can further damage the vascular endothelium (21).

Traditional office BP measurements do not adequately capture BP fluctuations over 24 h, especially in patients with high BPV. With advances in monitoring technology, 24-h ABPM is now more commonly used to assess short-term BPV. However, antihypertensive medications can influence BPV through various pathways. Calcium channel blockers, particularly amlodipine, have been shown to reduce BPV effectively, possibly by improving arterial compliance and baroreceptor sensitivity (22, 23). Diuretics may also lower BPV, though more evidence is needed (24). Sacubitril/valsartan, an angiotensin receptor-neprilysin inhibitor, has been shown to reduce BPV more effectively than valsartan alone (25). Therefore, these agents were consistently selected in this study to better manage BPV in the observation group while minimizing confounding errors arising from differences in medication choices.

Accurate BP assessment is essential for guiding antihypertensive therapy. While elevated BP exacerbates vascular damage, excessive BP reduction may lead to hypoperfusion in patients with impaired autoregulation. Lifestyle factors such as diet, emotional state, and physical activity also influence BP fluctuations. Clinicians should emphasize both BP and BPV monitoring to optimize treatment and achieve stable, long-term BP control within the normal range. In this study, BPV was measured 24 h after AIS onset in patients with stable pre-stroke BP who did not receive reperfusion therapy. Our results support the role of BPV as an independent risk factor for stroke severity and prognosis. Both NIHSS and mRS scores were positively correlated with BPV, indicating that patients with higher BPV experienced more severe neurological deficits and worse outcomes at 90 days. Importantly, patients whose BPV improved after tailored antihypertensive therapy showed better functional outcomes, suggesting that BPV reduction may confer clinical benefits.

Considering stroke recurrence is crucial when assessing the prognosis of a patient with AIS. No stroke recurrence was observed within 90 days, likely due to the small sample size and short follow-up period. Future studies with larger cohorts and longer follow-up are needed to assess the impact of BPV modulation on recurrence.

## Conclusion

Despite the small sample, our findings suggest that higher BPV is associated with worse 90-day outcomes in patients with AIS and hypertension. This finding implies that BPV monitoring may aid in risk stratification. However, randomized controlled trials are needed to establish causality. We recommend incorporating dynamic BP monitoring and BPV assessment into routine clinical practice for patients with AIS, particularly those with hypertension or normal mean BP.

### Limitations

The limitations of this study include its single center design, small sample size, and short follow-up period. The short follow-up period precluded further assessment of the relationship between BPV and recurrence of AIS. Therefore, larger multicenter studies with longer follow-up periods are needed to confirm these findings. The potential influence of confounding variables—notably medication adherence and lifestyle—cannot be excluded and may have biased the current results. Accordingly, more stringent control of these factors will be implemented in subsequent studies.

## Data Availability

The original contributions presented in the study are included in the article/Supplementary material, further inquiries can be directed to the corresponding authors.
